# Experimental Open Air Burning of Vegetation Enhances Organic Matter Chemical Heterogeneity Compared to Laboratory Burns

**DOI:** 10.1021/acs.est.3c10826

**Published:** 2024-05-22

**Authors:** Allison N. Myers-Pigg, Samantha Grieger, J. Alan Roebuck, Morgan E. Barnes, Kevin D. Bladon, John D. Bailey, Riley Barton, Rosalie K. Chu, Emily B. Graham, Khadijah K. Homolka, William Kew, Andrew S. Lipton, Timothy Scheibe, Jason G. Toyoda, Sasha Wagner

**Affiliations:** ∇ Marine and Coastal Research Laboratory, Pacific Northwest National Laboratory, Sequim, Washington 98382, United States; ‡ Department of Environmental Sciences, University of Toledo, Toledo, Ohio 43606, United States; § Pacific Northwest National Laboratory, Richland, Washington 99354, United States; ∥ Department of Forest Ecosystems and Society, Oregon State University, Corvallis, Oregon 97331, United States; ⊥ Department of Forest Engineering, Resources and Management, Oregon State University, Corvallis, Oregon 97331, United States; # Department of Earth and Environmental Sciences, Rensselaer Polytechnic Institute, Troy, New York 12180, United States; ○ Environmental Molecular Science Laboratory, Richland, Washington 99354, United States; ◆ School of Biological Sciences, Washington State University, Pullman, Washington 99164, United States; ● Center for Environmental and Stable Isotope Analysis, Rensselaer Polytechnic Institute, Troy, New York 12180, United States

**Keywords:** pyrogenic organic matter, charcoal, leachate, wildfire

## Abstract

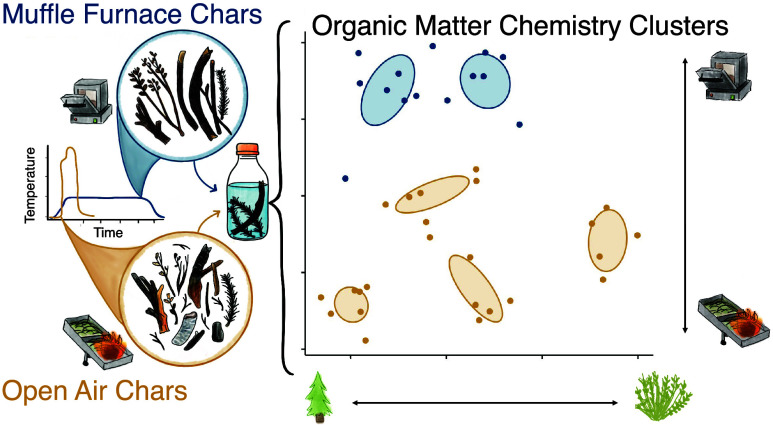

Wildfires produce solid residuals that have unique chemical and physical properties compared to unburned materials, which influence their cycling and fate in the natural environment. Visual burn severity assessment is used to evaluate post-fire alterations to the landscape in field-based studies, yet muffle furnace methods are commonly used in laboratory studies to assess molecular scale alterations along a temperature continuum. Here, we examined solid and leachable organic matter characteristics from chars visually characterized as low burn severity that were created either on an open air burn table or from low-temperature muffle furnace burns. We assessed how the different combustion conditions influence solid and dissolved organic matter chemistries and explored the potential influence of these results on the environmental fate and reactivity. Notably, muffle furnace chars produced less leachable carbon and nitrogen than open air chars across land cover types. Organic matter produced from muffle furnace burns was more homogeneous than open air chars. This work highlights chemical heterogeneities that exist within a single burn severity category, potentially influencing our conceptual understanding of pyrogenic organic matter cycling in the natural environment, including transport and processing in watersheds. Therefore, we suggest that open air burn studies are needed to further advance our understanding of pyrogenic organic matter’s environmental reactivity and fate.

## Abstract 2

Our study finds that the type of materials released from charcoals in laboratory studies is not reflective of what we may see in rivers after a wildfire.

## Introduction

Wildfires are known to transform the terrestrial landscape by consuming vegetation and soil organic materials and producing large amounts of greenhouse gas emissions and solid residues.^1^ These solid residues, known collectively as pyrogenic materials, often referred to as chars or charcoals, can release organic matter from terrestrial to aquatic ecosystems for decades after fires occur.^2,3^ Fire regimes are different across ecosystems,^4^ resulting in variable fire intensities that produce diverse quantities and qualities of pyrogenic materials. For example, vegetation from grassland and forested ecosystems produce distinct quantities and chemistries of pyrogenic materials.^5,6^ Transformed materials from wildfires, burning at a range of intensities, have unique physical and chemical properties compared to their unburnt counterparts.^3^ Even under similar burning conditions, differing fuel types often generate distinct modifications to organic matter released,^5^ complicating our understanding of that material’s fate in the environment.

Muffle furnace burning conditions are a popular choice to create chars in a controlled laboratory setting to study their chemical and physical properties, often used to better understand wildfire impacts on organic matter chemistry.^7−9^ This stems from the body of literature focused on understanding the transformations of organic matter in biochars, which are produced from vegetation or agricultural waste under limited oxygen conditions to create advantageous physical and chemical properties for nutrient amendments, pollution remediation, and other agricultural usages.^10,11^ Based on knowledge gained from muffle furnace burns, chemical transformations in chars occur from lower to higher temperatures.^6,12,13^ Chars produced from these types of experiments are more soluble if burned at lower temperatures^5,6,8,14^ compared to higher temperatures, leading to the currently accepted paradigm that chars produced under low-temperature combustion conditions generate larger quantities of leachable materials, which is relevant to understanding how the amount and composition of organic matter may shift post-fire.

In the environment, it is often rare to know the exact energy released from a fire (fire intensity) across an ecosystem that produces the chars left on the landscape, which influences ecosystem responses.^15^ Due to this, field studies often use visual characterization of chars and burned soils after the fire to determine potential organic matter loss, based on ash color, degree of consumption, and degree of char, cumulatively known as burn severity.^15,16^ Therefore, environmental chars that are visually characterized within a single burn severity category may result from a variety of fire intensities, complicating our understanding of their potential fate.^17^

Despite the widespread use of muffle furnace burns to simulate wildfire char from the field, there is an emerging body of literature suggesting that these materials are not universally chemically interchangeable.^18−20^ Oxygen availability is thought to be a key driver of organic matter transformations under pyrolysis/combustion and has been suggested to be a major difference between muffle furnace and natural charring conditions.^18^ However, direct comparisons between naturally derived and oven-created chars can be further complicated by differences in combustion due to the unknown temperature, fuel moisture, fuel density, and duration of heating that exists in the environment, all of which can influence organic matter chemistry after a burn.^15,21^ Relating charring temperature to burn intensity and, subsequently, burn severity is also extremely complex, and pairing molecular level information to these relationships remains underexplored.^22,23^

To combat these comparability issues, we simulated natural wildfire burning conditions on an open air burn table, where we were able to create predictable fire conditions by manipulating and quantifying fire behavior characteristics on a mesoscale. This may better mimic wildfire burning conditions by simulating field-relevant fuel loadings and oxygen conditions.^18^ After the burn experiments, we examined the resulting chars’ solid and leachable organic matter characteristics relative to low-temperature muffle furnace chars to assess how the methodological differences in combustion may manifest in unique dissolved and solid concentrations and compositions for chars visually characterized as low burn severity. We also explored the implications of these results on the potential environmental fate of the vegetation derived chars.

## Materials and Methods

Data sets used in this manuscript are openly available on the Environmental System Science Data Infrastructure for a Virtual Ecosystem (ESS-DIVE) repository, and data sets used in this study were retrieved from version 3 of Grieger et al.^24^ All code and data set manipulations used to generate results presented in this manuscript are also available on ESS-DIVE.^25^ Detailed methodology regarding vegetation collection, sample storage, and analytical methods can be found in the accompanying data set.^24^

### Vegetation Materials

Vegetation was collected to represent living vegetation and litter materials from fire-prone land cover types in the Pacific Northwest, including Douglas-fir forests (*Pseudotsuga menziesii*), mixed conifer forests (*Pseudotsuga menziesii* and *Pinus ponderosa*), ponderosa pine mountain woodlands (*Pinus ponderosa* and *Artemisia tridentata*), and sagebrush shrublands (*Artemisia tridentata*). For all land cover types, a mix of woody and canopy material was collected from the primary vegetation species present.^24^ Thus, the species listed in parentheses above are the only ones present in their respective land cover types.

### Burn Experiments

We completed experimental burns by manipulating burn temperature, duration of heating, fuel moisture content, fuel density, and vegetation conditions (i.e., living or litter) to create chars formed under variable fire behavior characteristics.^20,21^ The ratio of canopy to woody material was held constant within a land cover type for each burn. The temperature (°C) and duration (s) of heating were monitored with thermocouples (Omega Type K Thermocouple, Omega Engineering) during open air burns and with the oven’s thermocouple for muffle burns. In open air burns, the thermocouples were positioned at regular intervals within the fuels horizontally across the entire length of the burn tables (each was placed in the center of the fuels vertically). For the open air burns, a burn table was configured at a 5° angle, with metal barriers separating the land cover type treatments. Straw was used in the bottom most quadrant as a fire starter. Metal barriers were briefly lifted to allow flames to enter the above quadrant and then quickly replaced once lit. Grab samples were taken during each burn, with target temperature based on commonly used parameters from other studies, aimed to represent low (target ∼250–300 °C; actual 300 °C, labeled “Open Air 300”) and moderate/high (target ∼600 °C; actual 600 °C, labeled “Open Air 600”) temperature burns.^6,14^ Composite grab samples near the thermocouple reporting the target temperature were taken during the burns with metal tongs (cleaned with isopropyl alcohol before taking the sample). Burn experiments were ended when flames and smoldering ceased. Once cooled, a grab sample of material that was completely combusted (i.e., white to orange colored ash) was collected separately and any remaining char was homogenized and labeled as “Open Air End Char”. Each grab sample was homogenized in the laboratory before subsampling and analyses.

Burn temperature and heating duration of muffle furnace burns were based on commonly used parameters from other studies, aimed to represent low-temperature burns.^6,14^ Land cover type treatments were trimmed to fit inside a ceramic crucible with a lid. Three replicate muffle burns were conducted for each land cover type treatment. A Thermo Scientific Thermolyne F48000 benchtop muffle furnace was used with a ramp up of 30 °C/min from 25 to 250 °C. Temperature was held at 250 °C for 1 h.

### Burn Severity Classification

Burn severity, the resultant impact of burning intensities that can experience a range of temperatures and durations,^15^ was visually determined on all chars postburn and was based on ash color, degree of consumption, and degree of char following US Forest Service field metrics for determination of soil burn severity.^16^ Only chars classified as low burn severity were used herein. Low burn severity was visually characterized as little to no change from prefire status, with recognizable fine fuels (needles and leaves) present and less than 50% consumption of litter materials/some char present, with needles and leave structures charred, yet mostly intact.^16^ Solid chars were dried and stored in the dark at room temperature and were well ventilated until further analysis.

### Leaching Experiments

Unground chars were weighed in triplicate for leaching experiments,^24^ where 1 L of synthetic rainwater (detailed prep outlined in the “BSLE_Laboratory_Protocol” file in the methods folder of Grieger et al.^24^) was added to 25 g of char and shaken in the dark at 25 °C. Briefly, artificial rainwater was prepared with ionic concentrations found in rainwater in the Pacific Northwest, excluding any carbon or nitrogen containing compounds. After 24 h of mixing, the leachate was sequentially filtered through a 2 mm × 0.6 mm PTFE mesh, a precombusted nominal 0.7 μm GF/F filter, and then a 0.2 μm Gamma irradiated filter. Aliquots were taken and stored in the dark at 4 °C until further processing.

### Solid Char Chemistry

Representative subsamples from the chars produced were finely ground with a ball mill and used for subsequent analyses. Total carbon (C) and nitrogen (N) were determined using an elemental analyzer (ECS 8020; NC Technologies, Italy). Solid-state cross-polarization (CP) nuclear magnetic resonance (NMR) experiments were performed at 11.7 T (500.18 MHz for ^1^H and 125.78 MHz for ^13^C) on an Agilent VNMRS spectrometer at the Environmental Molecular Sciences Laboratory (EMSL, Richland, WA) using a 4 mm MAS HXY probe from Revolution NMR tuned to ^1^H/^13^C. Carbon chemical shifts were referenced to a secondary standard of the methylene peak of adamantane at 38.48 ppm relative to tetramethylsilane (TMS) at 0 ppm. Sample spinning speed was 10 kHz, and RF field strengths used for CP^26^ were calibrated at 45 kHz for ^1^H and 35 kHz for ^13^C with a 1 ms contact time with a ramp^27^ on the ^1^H spin lock. The ^1^H decoupling scheme used was SPINAL-64^28^ with a field strength of approximately 42 kHz. NMR spectra were analyzed after scaling to the sample mass. Integral regions were summed and binned using the nmrrr R package,^29^ using the functional groups assignments by Clemente et al.;^30^ the table of group assignments is included in the R package.^31^ The percentage of aromaticity was calculated using the following equation:^32,33^
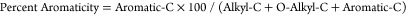
1

### Leachate Chemistry

Dissolved organic carbon (DOC) and total dissolved nitrogen (TDN) were simultaneously measured on a Shimadzu TOC-L Total Organic Carbon Analyzer in precombusted amber vials within a week of leaching and filtering. Leached C and N in mg g C^–1^ were calculated as in eq 1 of Fischer et al.:^34^
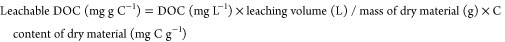
2

The distribution coefficients of C concentrations between the solid and aqueous phases were calculated as in equation 1 of Myers-Pigg et al.:^35^
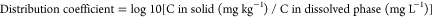
3

Simultaneous absorbance and 3D Excitation Emission Matrices (EEMs) were measured on a HORIBA Aqualog optical spectrometer with samples diluted to a standard concentration of 5 mg of C L^–1^. Corrected absorbance and EEMs were normalized to native DOC concentration, and common optical indices were derived.

Solid phase extraction (SPE) was used to clean up and concentrate the leachates^36^ for Fourier transform ion cyclotron resonance mass spectrometer (FTICR-MS) and benzene polycarboxylic acid (BPCA) measurements. A loading ratio of DOC to PPL sorbent of 1:45 was used for all SPE extractions.^36,37^ A 21T FTICR-MS was used to collect high-resolution mass spectra at EMSL.^38^ Leachate SPE extracts were directly injected, ionized with electrospray ionization (ESI), and acquired in negative mode. 450 scans were collected across 150–1000 *m*/*z* that were internally calibrated. Chemical formulas of C, H, O, N, S, and/or P were assigned on peaks with a S/N over 2 and mass measurement error <0.5 ppm using Formularity.^39^ The modified aromaticity index (AI_mod_) was calculated as in Koch and Dittmar.^40,41^

Complete methodological details for the quantification of BPCAs as a proxy for highly condensed aromatic structures (e.g., dissolved black carbon, DBC) are provided in Barton and Wagner and Wagner et al.^42,43^ Briefly, a small amount of dried methanol SPE extract was thermochemically oxidized with nitric acid at 160 °C for 6 h. Individual penta- (B5CA) and hexa- (B6CA) substituted BPCAs were separated via high performance liquid chromatography (HPLC) and quantified using a diode array detector. Final DBC concentrations were calculated from the following power-relationship:^44^
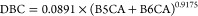
4

The BPCA ratio was further calculated as B6CA/B5CA and serves as an indicator of increasing degree of DBC polycondensation with increasing B6CA/B5CA ratio.^43,45^

### Statistics

All statistical tests were conducted in R version 4.2.1^46^ using RStudio version 2022.07.2. Data sets were tested for normality (using Shapiro-Wilk’s test) and equal variance (using Bartlett’s test) before statistics were performed; when these assumptions were not met, appropriate normalizations were determined using the bestNormalize package^47,48^ and the transformed data sets were retested for normality. Tukey post hoc analyses were conducted using least-squares means of the model fit using emmeans.^49^

To explore how muffle furnace and open air burning conditions would generate char concentrations that might differ by land cover types, differences in solid char chemistry were assessed by land cover type, burning type, and their interactions by two-way analysis of variance (ANOVA); rank normalized transformation was performed prior to conducting solid char chemistry ANOVAs.^50^

The relationship between burn type, land cover type, and dissolved organic matter concentrations in the leachates was explored through mixed effects models (using R package lme4^51^) with land cover type and burn type as fixed effects and leachate replicate as a random effect. Multicollinearity of fixed effects was inspected using the variance inflation factor (VIF) in the R package car.^52^

To explore the relationship among burn type, land cover type, and metrics of aromatic C, we performed a Spearman correlation matrix in R on the metrics of aromaticity determined in the leachates and chars (% aromaticity in ^13^C NMR spectra,^32,33^ specific UV absorbance at 254 nm,^53^ modified aromaticity index from FTICR-MS data sets,^40,41^ and the ratio of B6CA/B5CA^43^).

To assess chemical differences from muffle furnace and open air burns across land cover types, we clustered samples into groups using composition data (absorbance, fluorescence, high-resolution mass spectrometry, and NMR) and total concentrations of C and N of the chars and leachates from muffle and open air burns only. Before performing any clustering analyses, data sets were mean centered and scaled (using the scale function in R). Data sets were examined by using K-means clustering. The number of clusters chosen were informed with elbow, silhouette, and gap statistic methods (using the cluster package^54^).

## Results and Discussion

### Open Air Burning Creates Variable Intensity Fires

**Figure 1:**
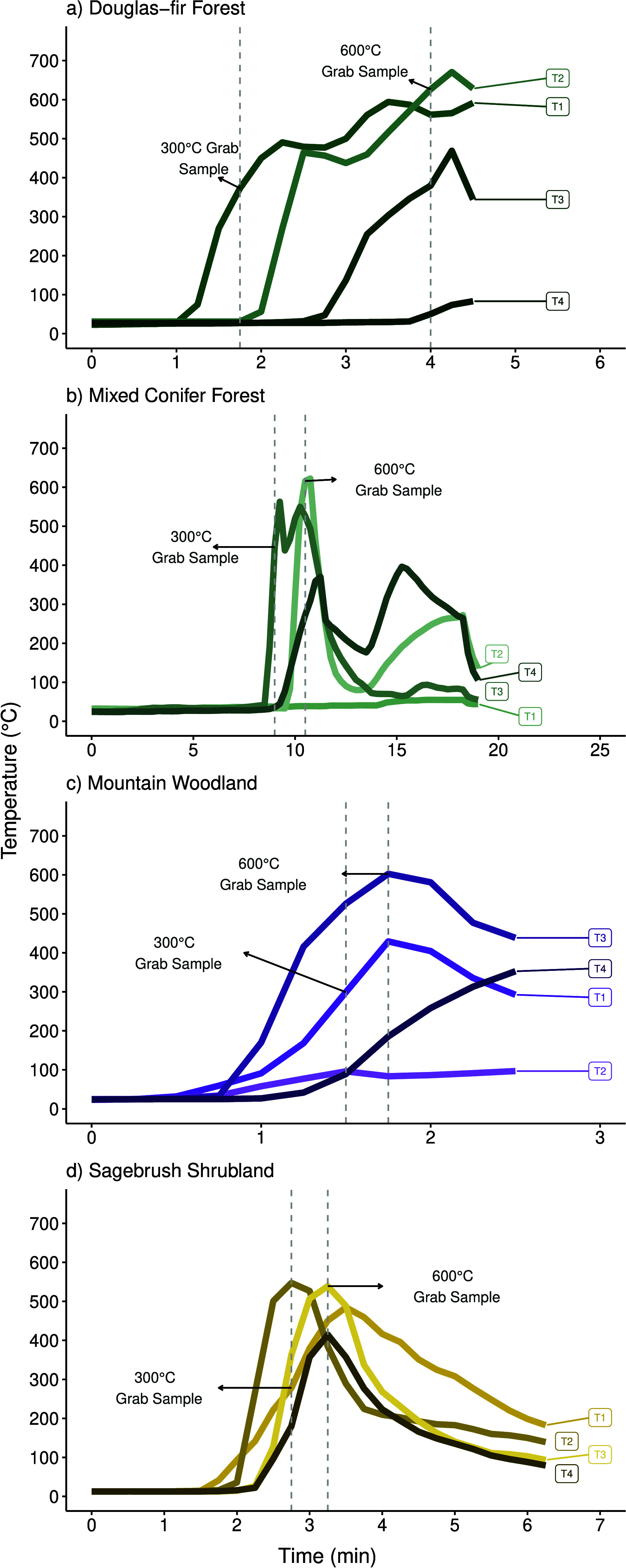
Example temperature profiles for each land cover type (a–d). T1–4 are individual thermocouples that were distributed across the burn table. Temperature grab samples (300 and 600 °C) were collected next to the thermocouple at the target temperature, when the specific thermocouple reached that temperature. Note variable *x* axes. Complete temperature profiles for all samples can be found in Grieger et al.^24^

The same visual burn severity classification experienced a range of maximum temperatures and burn durations during the open air burns, while the muffle furnace burns experienced identical burning conditions for each of the treatments. Therefore, burn severity was not exclusively related to temperature but was also related to the duration of heating and source vegetation across our open air burns. All open air burns achieved an open flame during the experiment. The open air burns experienced a range in burn profiles (Figure 1), with the maximum temperature and duration of heating dependent on the land cover type and experimental burning conditions. The open air burns ranged from <1 min to almost 20 min, and max temperature ranged from ∼270 to ∼630 °C (Table S1). In contrast, all muffle furnace burns were held to a standard temperature of 250 °C and duration of 60 min. The differences in the burning conditions may have resulted in varying degrees of oxygen availability across the treatments and fuel particles during the burns. While we did not have the ability to monitor oxygen content during these burns, estimates of mass loss in the open air burns were higher than muffle furnace burns, indirectly supporting variation in oxygen conditions experienced in the different treatments (Table S2). Oxygen availability has been previously correlated with char C content, with increasing C content present with decreasing oxygen.^7,20^

### Variable Combustion Conditions Influence Solid Char Chemistry

**Figure 2:**
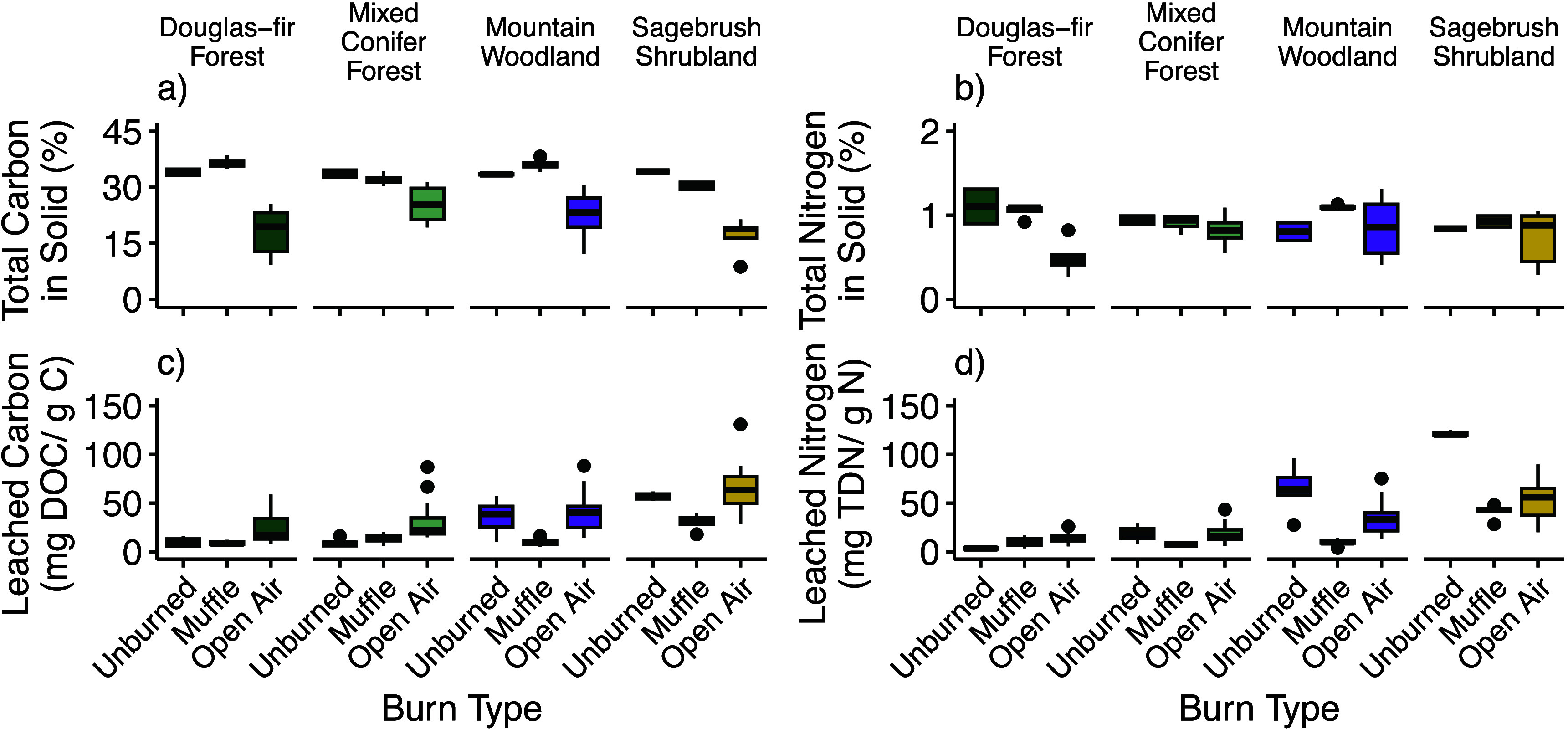
Total % C and N in solid chars. Leachability of C and N is higher in the open air burns compared to the muffle burns across plant types. Leachability of C and N in the unburned plant material varies by land cover type. The number of observations in each category across each burn type is available in Myers-Pigg et al.^25^

Two-way ANOVA indicated that the differences in percent C were significant among unburned, muffle, and open air chars (*p* < 0.001, *F* = 80.298) and land cover (i.e., Douglas-fir forest, mixed conifer forest, mountain woodland, and sagebrush shrubland; *p* < 0.001, *F* = 8.469), with a significant interaction between burn type and land cover type (*p* < 0.01, *F* = 3.432) (Figure 2). Open air burning created chars with significantly different percent C than both muffle furnace burns and unburned plants. Among different land cover types, there were no significant differences between the unburned and muffle furnace solid percent C (Tukey post hoc analysis; *p* = 0.834). The solid char C and N concentrations of the muffle furnace burns were more similar to that of the unburned plant material compared to the open air burns within a land cover type. Differences in percent N were significant by burn type only (two-way ANOVA; *p* < 0.01, *F* = 6.991) (Figure 2). Post hoc tests revealed that the observed difference by burn type for percent N was driven by significant differences between the muffle and open air chars only (*p* < 0.001).

Total percentages of C and N from all land cover types were less in the open air burns compared to the muffle furnace burns; however, this trend was more pronounced in total C from land cover types with just a single species compared to those with mixed species present (Figure 2). This suggests a more varied change in C and N concentration post-fire in ecosystems with larger diversity in vegetation species compared to those dominated by a single species. Therefore, differences in land cover type result in charred materials upon burning that vary in their susceptibility to biological degradation in the environment.^55,56^

**Figure 3:**
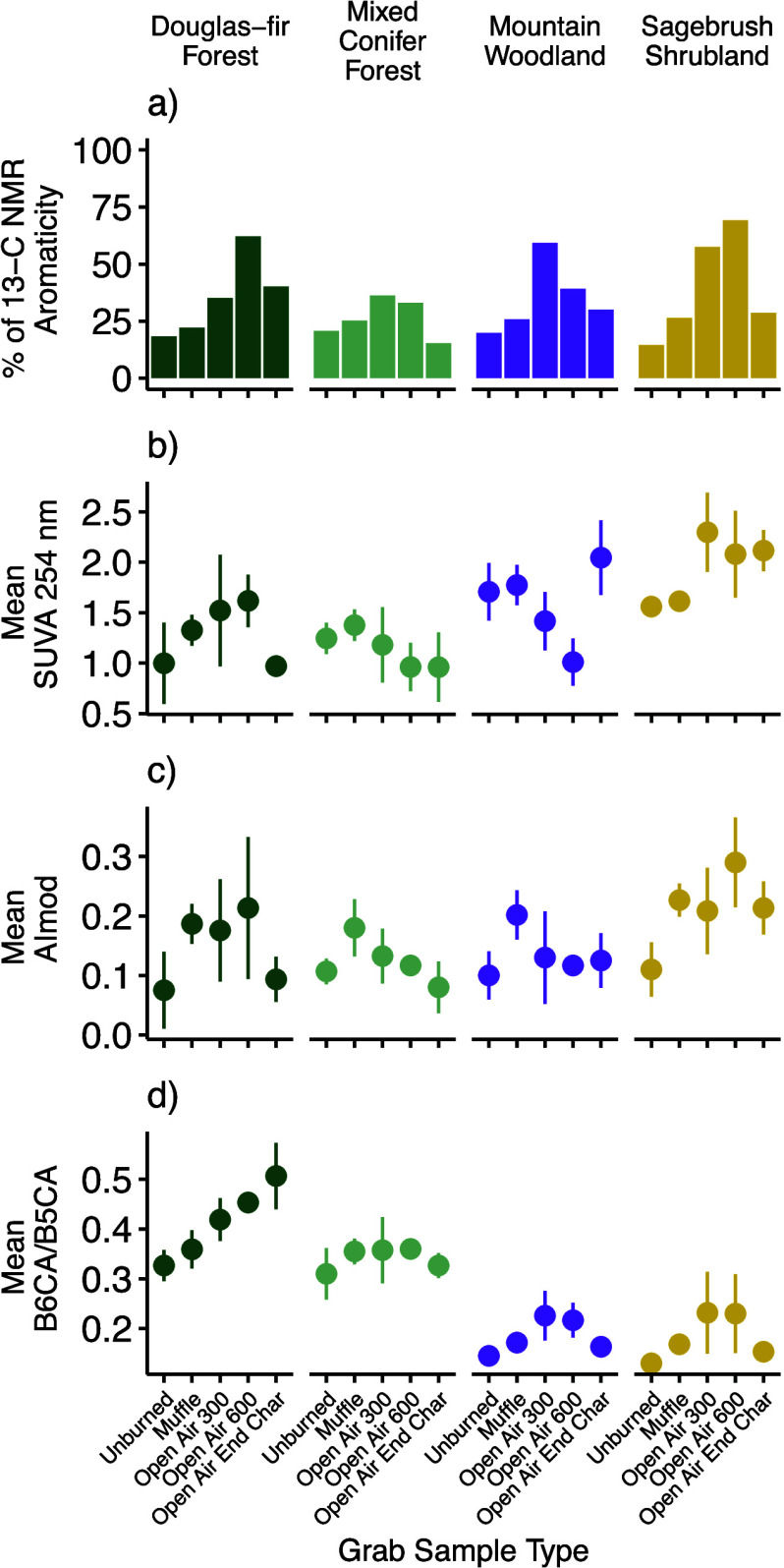
Chemical metrics assessing aromaticity in the solids, represented in the bars (% of ^13^C NMR aromaticity) and leachates, represented in points with lines indicating standard deviation across replicate leachates (SUVA, AImod, B6CA/B5CA). Aromaticity metrics vary by grab sample type and land cover type.

There is a difference in the chemical composition of the C in the solid char samples, which varies with land cover type most notably in the open air chars (Figure S1). Anomeric C decreased for all land cover types and burn types compared to the unburned vegetation, while aromatic C increased in both muffle and open air burns, except for the mixed conifer open air chars that remained at the end of the burn (i.e., end chars). Alkyl C increased in muffle furnace burns for all land cover types but exhibited little change, losses, or gains in the relative percentage of C in open air burns compared to the unburned materials. For all land cover types, the relative proportion of aromatic C was the highest and the relative proportion of O-alkyl C was the lowest in the 300 and 600 °C grab samples, relative to muffle furnace burns or chars that remained at the end of the burn (i.e., end chars), compared to the unburned materials (Figure S1). Cumulatively, these results show a higher percentage of aromaticity as calculated by ^13^C NMR spectra^32,33^ in the solid chars in the 300 and 600 °C grab samples compared to the unburned, muffle, or chars that remained at the end of the burn (i.e., end chars) (Figure 3). Contrary to studies spanning visual burn severity^17^ or temperature gradients,^20,57^ we did not see systematic losses of O-alkyl C and systematic increases in aromatic C across grab samples collected as the burn reached specific temperatures during the open air burns, highlighting extreme chemical complexity that results from variable burn intensities that can occur within a single visual burn severity classification.

The relationship between char aromaticity and burn temperature has been proposed from laboratory experiments as a “molecular thermometer”.^19^ While we do find a positive linear relationship between the percentage of aromaticity observed via solid-state ^13^C NMR in the solid chars with maximum burn temperature, this relationship is relatively weak and does not directly support the molecular thermometer notion (Pearson’s *r* = 0.474), as nonlinearities occur across open air temperature grabs (Figure S2). This is most notable in the land cover types that contain sagebrush (Figure S2) and suggests a more nuanced relationship between aromaticity and burn temperature for char produced in open air.

The availability of oxygen may create higher degrees of condensation in shorter periods of time,^7,18^ increasing the char aromaticity. Previous research suggests that there is a relationship between char aromatization and oxygen conditions during burning.^13,19,58^ Solid-state ^13^C NMR results support this prior work and showed a higher percentage of aromatic functional groups in 300 °C open air chars, which experienced shorter burn time, than 250 °C muffle furnace chars, which experienced longer burn time (Figures S1 and S2). The aromaticity was also more homogeneous across land cover types in the muffle furnace burns than the 300 °C open air chars, which varied dramatically with land cover type (Figure 3). A weak negative linear relationship (Pearson’s *r* = −0.331) between duration of heating and percent aromaticity exists across the open air grabs (Figure S3). Duration of heating has been correlated to char chemical characteristics and has been suggested as the driver for the general lack of transferability between muffle furnace burns and field studies.^32^ Therefore, post-fire visual burn severity metrics may also need to be combined with fire behavior characteristics (such as fire rate of spread) and burn intensity metrics to develop a more holistic understanding of the most representative chemical mosaic that may exist across a landscape post-fire.

### Leachability of Muffle Furnace Chars Is Less than Open Air Burns, Impacting Observed Leachate Organic Matter Composition Across Low-Severity Burns

Across the land cover types, muffle furnace burns displayed less leachable C and N per gram of C or N in the chars compared with open air burns (Figure 2). While muffle furnace studies show that leachability of many dissolved phase elements is highest in lower temperature chars,^8,59,60^ we did not find congruence between low temperature muffle furnace char leachability and open air char leachability. In fact, across all land cover types, muffle furnace chars leached significantly less C than open air burns (Tukey post hoc analysis of linear mixed-effects model; *p* < 0.0001; Figure 2c). This may be due to relatively less soluble organic matter moieties produced in the muffle burns compared with the open air burns, which may be linked to oxygen availability. The difference in the amount of total dissolved N leached per gram of N in solid char between the muffle furnace burns was also significant (Tukey post hoc analysis of linear mixed-effects model; *p* = 0.0151; Figure 2d). We can also consider this finding using the distribution coefficients, which examine the relative concentrations of C in the solid and aqueous phases (also known as a partition coefficient; the higher the coefficient value, the less soluble it is in water). Understanding experimental distribution coefficients may be useful as an input parameter of including pyrogenic C cycling into fate and transport models (e.g., similar to development within the contaminant literature^61^). We find that the distribution coefficients of C from the muffle furnace chars are higher than that of the open air chars (Figure S4), supporting the conclusion that muffle furnace chars are less soluble than their open air counterparts. Estimates of pyrogenic C phase distribution in environmental samples is relatively consistent across the few studies that examine pyrogenic C in the dissolved and particulate phases.^35^ This is regardless of the biomarker used to estimate the pyrogenic C content (low or high temperature markers, e.g., Table 1 in ref (35)). However, in muffle furnace studies, there is an increase in the distribution coefficients with increasing temperature.^8^ These contrasting observations between natural wildfires and burning in muffle furnaces support the idea that there are likely other constraints on the phase distribution of pyrogenic C in the environment, such as interaction with soils, minerals, and the hydrological cycle.^62^ Together, these results suggest that we may be underestimating the amount of soluble C and N released into the environment during rain events following fires that produce low severity landscape alterations by applying our understanding of muffle furnace chars for inferences made on the landscape scale, hindering our ability to accurately predict and quantify the downstream implications of wildfires on C cycling.

Leachate chemistry is not clearly linked to the differences observed in the solid chars (Figure 3; Table S3), supporting previous muffle furnace-based research that finds pyrogenic dissolved organic matter is chemically dissimilar to the char it was produced from.^8^ For example, the relationship between SUVA and AI_mod_ in the leachates and the aromaticity observed via NMR in the solid chars was highly variable (Table S3). Therefore, higher aromaticity in the solid chars is not directly translated to increased aromaticity in the leachates. This may be due, in part, to the relative solubilization of different chemical moieties produced during incomplete combustion (e.g., more soluble moieties will be flushed from the solid chars first) and may also relate to the observed differences in leachable C observed.

The shifting relationship in four common metrics of aromaticity measured among the different grab samples (Figure 3) highlights that the relationships between metrics of assessing aromaticity may be unique across chars visually assessed as low burn severity. These chars are produced under different temperature and duration of heating metrics, complicating the usage of certain metrics of aromaticity as proxies of other metrics of aromaticity in the environment, for example, using absorbance measurements as a proxy for degree of aromaticity measured by BPCAs.^44^ The relationships between the different metrics assessing aromaticity vary with combustion conditions (Table S3). There were strong Spearman’s correlations between SUVA and AI_mod_ from all land cover types for the leachates from 300 and 600 °C grab samples of the open air treatments; the mixed land cover types had the lowest SUVA and AI_mod_ values within those grab sample types. Interestingly, while there was a strong negative correlation between the muffle and open air 300 °C solid char aromaticity via NMR and the leachate B6CA/B5CA ratios, this was not observed in the open air 600 °C grab samples and the end chars. A strong positive correlation across these metrics with temperature was expected as BPCA condensation has been proposed as a possible molecular thermometer^19^ and generally increases along traditional temperature continuums in both solids and leachates.^8,13^ Thus, such differences have implications on our understanding of the fate and transport on the continuum of organic matter composition after a fire and as an ecosystem recovers and would benefit from additional study.

Nuances in our understanding of the chemical heterogeneity that exists within a burn severity category are important to advance our conceptual understanding of pyrogenic organic matter cycling in the natural environment. Burn severity is often used to assess post-fire alterations to the landscape in field-based and modeling studies, yet muffle burns are the most commonly used method to assess alterations along a combustion continuum at the molecular scale.^8^ Based on the multiparameter organic matter composition (based on absorbance, fluorescence, high-resolution mass spectrometry, and NMR data) of char and leachates herein, muffle furnace burns are clustered more closely to each other than open air burns, across land cover types (Figure S5). We suggest that chemical heterogeneity that exists among open air char solids and leachates is not well represented by muffle furnace burns produced from more homogeneous temperature and oxygen conditions. Consequently, the total concentration and organic matter composition of muffle furnace burns are not representative of open air burns at low severities. Similar to studies that examine soil burn severity metrics and soil organic matter chemical characteristics,^17,22,23^ mapping the shifts in dissolved organic matter chemistry that occur across wildfire burn severities from a variety of land cover types will be important to ascertain how shifting dissolved phase chemistries relate to differences in burn severity and their implications for downstream organic matter cycling.

### Land Cover Type May Influence Environmental Fate of Chars Produced from Low Severity Burns

The amount of material leached from low burn severity litters varied by vegetation materials from different land cover types. The lowest distribution coefficients were present in the sagebrush shrublands. This led to the highest amount of C leached into the aqueous phase of all the studied land cover types from sagebrush shrublands (Figures 2 and S4), which may be due to forested land cover types containing more insoluble C moieties, such as structural lignocelluloses.^63^

In the combustion continuum paradigm,^3^ where shifts in chemical composition are related to its thermal alteration along a combustion continuum, land cover type may be the most influential controlling factor on solid and dissolved phase pyrogenic C production at low temperatures because chemical moieties are more similar to the starting materials than highly condensed molecules produced at higher temperatures.^3,6,19^ In the burn severity paradigm,^16^ where consumption of organic matter is measured, increasing burn severity classifications are assessed through increasing consumption and charring of organic matter.^17,22,23^ While lower burn severities may contain a variety of pyrogenic materials thought to be derived from across the traditional combustion continuum,^3^ land cover type may exert control on the organic matter composition observed in low burn severity assessments, their leachability, and their environmental cycling. For example, organic matter composition of litter from partially burned soils vary by litter type, which has been previously linked to the chemical composition of the starting plant material.^64^ The concentration, distribution, and degradation of pyrogenic molecular markers and organic matter also depend on plant material type.^5,14,65,66^

This work highlights that chemical heterogeneities exist within a single burn severity category and a high solubility of C and N from low burn severity chars, potentially influencing our conceptual understanding of pyrogenic organic matter cycling in the natural environment. Fires burn the landscape heterogeneously, resulting in a mosaic of severities, though a proportion of high-severity wildfire is increasing in many ecosystems.^67,68^ As fire regimes continue to shift, land cover types in many regions may eventually shift from forested biomes to more shrublands, which could also alter C and nutrient storage and cycling in such ecosystems.^69^ Thus, vegetation-fire feedbacks, such as successional dynamics or shifts, may notably influence aquatic biogeochemical cycles, further highlighting areas of research needed to advance our understanding of wildfires on aquatic biogeochemical cycling.^1^

## Data Availability

All original data are available in Grieger et al.;^24^ all code and processed data used to support this manuscript are available in Myers-Pigg et al.^25^

## Supporting Information Available

Range in burn duration and max temperature for each burn by land cover type; mean and standard deviation of mass loss by land cover type and burn treatment type; Spearman’s correlation matrix by grab sample type; relative abundance of NMR functional group assignments for each char type; linear regression of max temperature and percentage of aromaticity determined by ^13^C-NMR; linear regression of duration of heating during open air burns and percentage of aromaticity determined by ^13^C-NMR; partition coefficients for each char type; K-means cluster analysis of char and leachate chemistries and concentrations (PDF)
