# Supplementary material for: Experimental Open Air Burning of Vegetation Enhances Organic Matter Chemical Heterogeneity Compared to Laboratory Burns
Source: Environ Sci Technol. 2024 May 22;58(22):9679–88. doi: 10.1021/acs.est.3c10826 (PMC11155678; doi:10.1021/acs.est.3c10826)
Supplement: Supplementary file 1 — es3c10826_si_001.pdf [file es3c10826_si_001.pdf]

Supporting Information for: Experimental open air burning of vegetation enhances organic matter chemical heterogeneity compared to laboratory burns

Allison N. Myers-Pigg[1,2*], Samantha Grieger[1], J. Alan Roebuck, Jr.[1], Morgan E. Barnes[3], Kevin D. Bladon[4,5], John D. Bailey[5], Riley Barton[6,7], Rosalie K. Chu[8], Emily B. Graham[3,9], Khadijah K. Homolka[1,†], William Kew[8], Andrew S. Lipton[8], Timothy Scheibe[3], Jason G. Toyoda[8], Sasha Wagner[6,7]

AUTHOR ADDRESS

[1] Pacific Northwest National Laboratory, Marine and Coastal Research Laboratory, Sequim, WA

[2] University of Toledo, Department of Environmental Sciences, Toledo, Ohio, USA

[3] Pacific Northwest National Laboratory, Richland, WA

[4] Department of Forest Ecosystems and Society, Oregon State University, Corvallis, OR

[5] Department of Forest Engineering, Resources and Management, Oregon State University, Corvallis, OR

[6] Department of Earth and Environmental Sciences, Rensselaer Polytechnic Institute, Troy, NY

[7] Center for Environmental and Stable Isotopes Analysis, Rensselaer Polytechnic Institute, Troy, NY

[8] Environmental Molecular Science Laboratory, Richland, WA

[9] Washington State University, School of Biological Sciences, Pullman, WA

[*] Corresponding Author

Supporting Information – pages S2-S5

Supporting Table S1 – page S2
Supporting Table S2 – page S2
Supporting Table S3 – page S2
Supporting Figure S1 – page S3Supporting Figure S2 – page S3
Supporting Figure S3 – page S4
Supporting Figure S4 – page S4
Supporting Figure S5 – page S5

Table S1. Range in burn duration and max temperature for each burn by land cover type.

Land Cover Type	Burn Treatment	Range in Burn Duration (minutes)	Range in Max Burn Temperature (°C)
Douglas-fir Forest	Open Air	1.1 - 17.6	295.3 - 626.5
	Muffle	60	250
Mixed Conifer Forest	Open Air	0.9 - 12.9	337.5 - 615.9
	Muffle	60	250
Sagebrush Shrubland	Open Air	0.8 - 3.4	278.3 - 574.4
	Muffle	60	250
Mountain Woodland	Open Air	1.2 - 11.4	279.6 - 634.5
	Muffle	60	250

Table S2. Mean and standard deviation of mass loss by land cover type and burn treatment type.

Burn Treatment	Land Cover Type	Mean Mass Loss (%)	Standard Deviation Mass Loss (%)
Muffle	Douglas-fir Forest	26.72	3.71
	Mixed Conifer Forest	28.56	10.70
	Mountain Woodland	25.66	3.38
	Sagebrush Shrubland	30.02	3.73
Open Air	Douglas-fir Forest	77.17	6.77
	Mixed Conifer Forest	74.94	8.33
	Mountain Woodland	65.75	15.51
	Sagebrush Shrubland	81.87	4.39

Table S3. Spearman's Correlation Matrix by Grab Sample Type

Grab Sample Type		Solid NMR Aromaticity	Leachate SUVA	Leachate B6CA/B5CA	Leachate AImod
Unburned	Solid NMR Aromaticity	1.0	0.0	0.4	-0.2
	Leachate SUVA	0.0	1.0	-0.8	0.4
	Leachate B6CA/B5CA	0.4	-0.8	1.0	-0.8
	Leachate AImod	-0.2	0.4	-0.8	1.0
Muffle	Solid NMR Aromaticity	1.0	0.8	-1.0	0.8
	Leachate SUVA	0.8	1.0	-0.8	0.6
	Leachate B6CA/B5CA	-1.0	-0.8	1.0	-0.8
	Leachate AImod	0.8	0.6	-0.8	1.0
Open Air 300	Solid NMR Aromaticity	1.0	0.0	-1.0	-0.4
	Leachate SUVA	0.0	1.0	0.0	0.8
	Leachate B6CA/B5CA	-1.0	0.0	1.0	0.4
	Leachate AImod	-0.4	0.8	0.4	1.0
Open Air 600	Solid NMR Aromaticity	1.0	1.0	0.0	0.9
	Leachate SUVA	1.0	1.0	0.0	0.9
	Leachate B6CA/B5CA	0.0	0.0	1.0	0.2
	Leachate AImod	0.9	0.9	0.2	1.0
End Char	Solid NMR Aromaticity	1.0	0.2	0.4	0.2
	Leachate SUVA	0.2	1.0	-0.8	1.0
	Leachate B6CA/B5CA	0.4	-0.8	1.0	-0.8
	Leachate AImod	0.2	1.0	-0.8	1.0

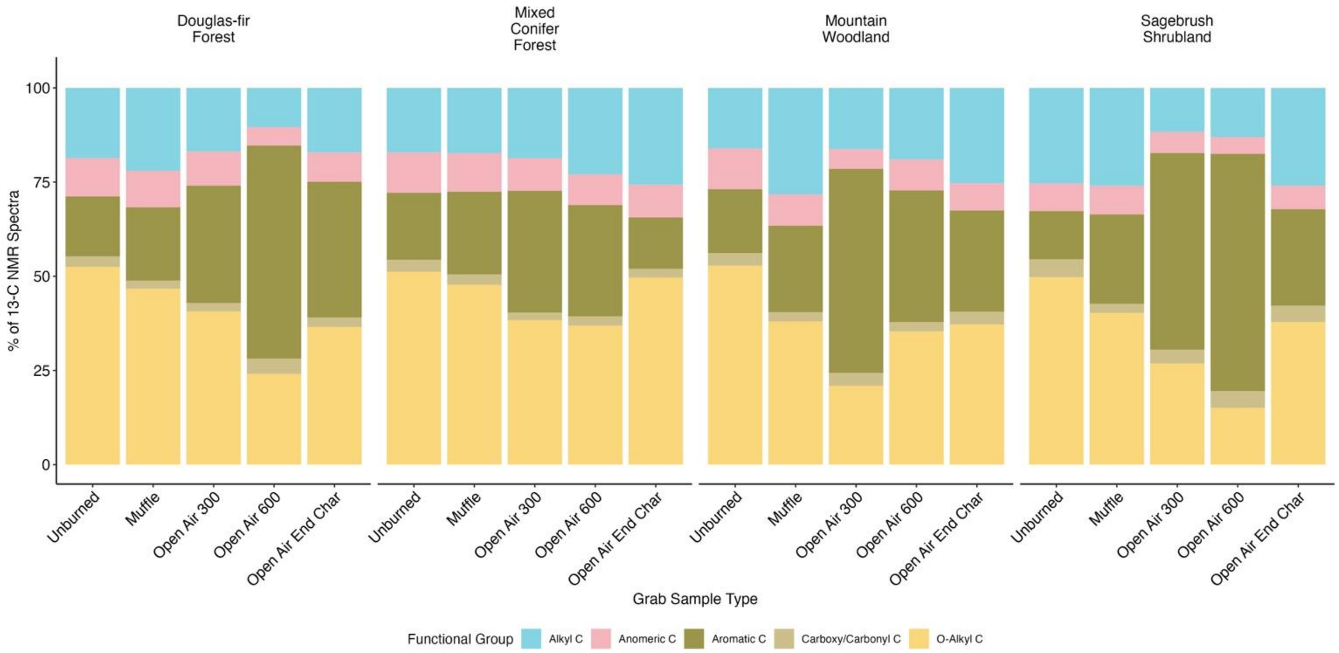

Figure S1. Relative abundance of NMR functional group assignments for each char type. The underlying data of relative abundances is available in Myers-Pigg et al [25].

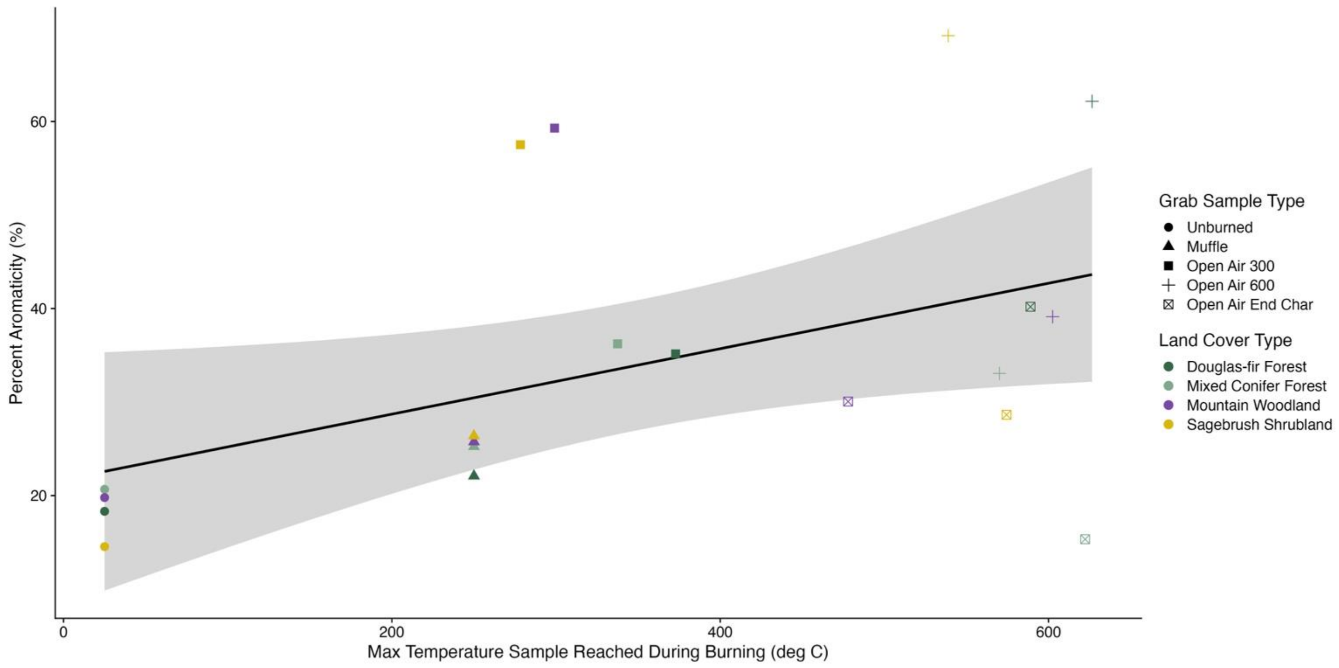

Figure S2. Linear regression of max temperature and percentage of aromaticity determined by ^{13}C-NMR.

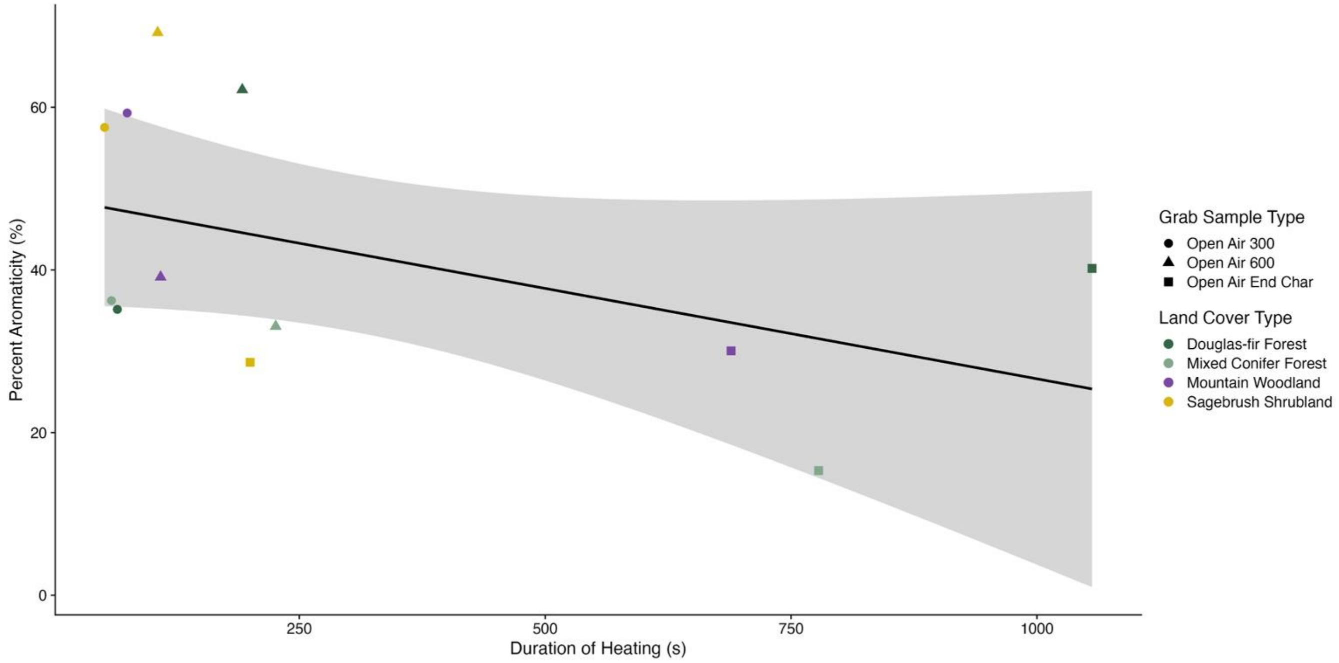

Figure S3. Linear regression of duration of heating during open air burns and percentage of aromaticity determined by ^{13}C-NMR.

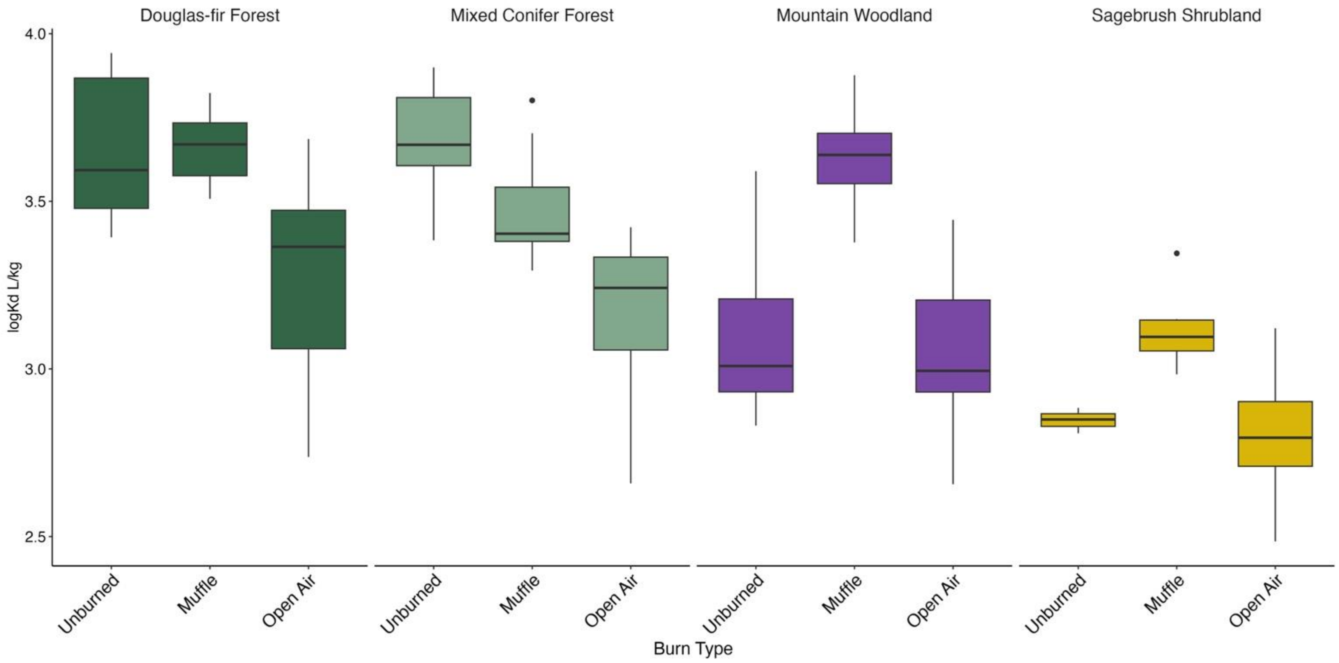

Figure S4. Partition coefficients (log Kd) for each char type. Open air boxplots contain all open air samples within each land cover type. The number of observations in each category across each burn type is available in Myers-Pigg et al [25].

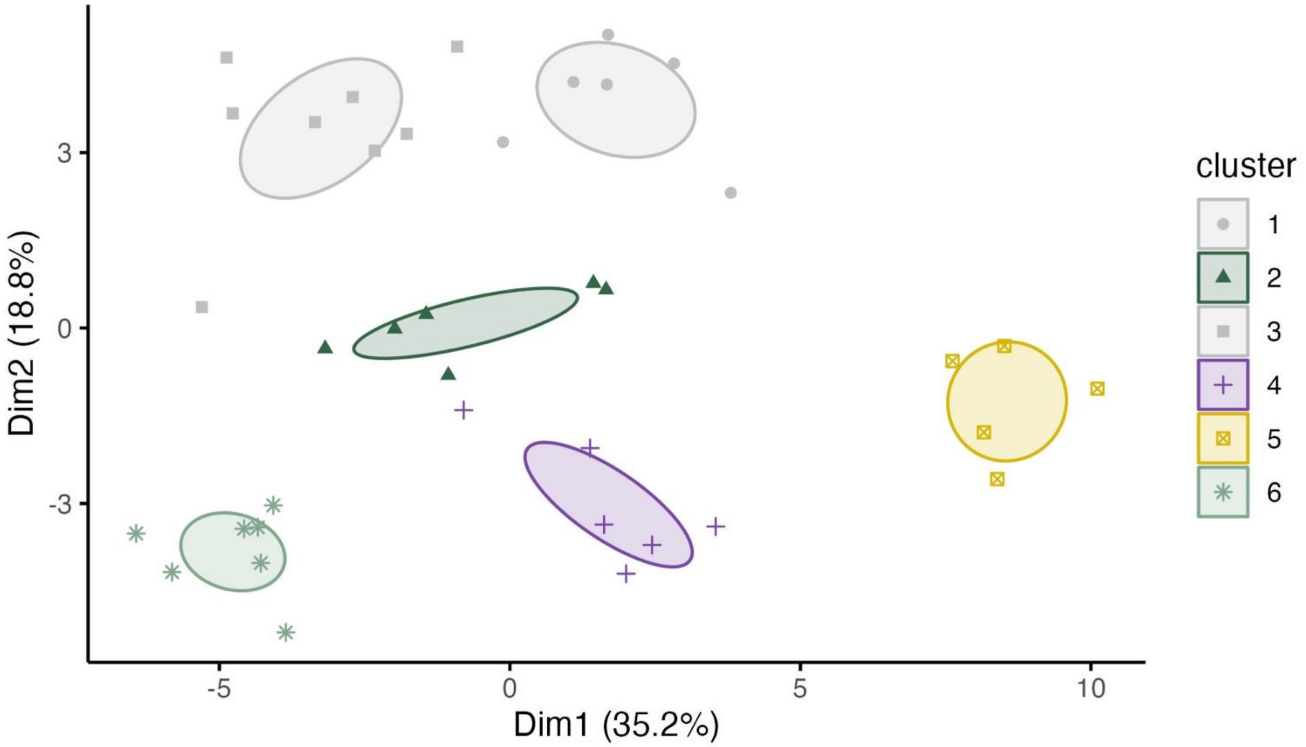

Figure S5. K-means cluster analysis of char and leachate chemistries and concentrations. Samples cluster by land cover type and burn severity/burn type (muffle, open air). Cluster 1: muffle sagebrush shrubland, cluster 2: open air Douglas-fir forest, cluster 3: muffle Douglas-fir and mixed conifer forests, cluster 4: open air mountain woodland, cluster 5: open air sagebrush shrubland, cluster 6: open air mixed conifer.
